# Electric Current Detection Based on the MR Signal Magnitude Decay

**DOI:** 10.1002/mrm.29278

**Published:** 2022-05-05

**Authors:** Igor Serša

**Affiliations:** ^1^ Jožef Stefan Institute Ljubljana Slovenia; ^2^ Institute of Pathophysiology, Faculty of Medicine University of Ljubljana Ljubljana Slovenia

**Keywords:** electric current detection, gradient‐echo imaging, current‐induced magnetic field gradient, signal magnitude decay

## Abstract

**Purpose:**

Conventional current density imaging method, which relies on the detection of the magnetic field induced by the current in an image phase, is demanding and difficult to perform. In this study, a much simpler signal‐magnitude‐decay (SMD)–based current detection method is proposed.

**Methods:**

Conductive test and biological samples were imaged at various TE times using the gradient‐ or spin‐echo imaging sequences with superimposed constant or bipolar currents, respectively. The SMD curve was sampled for each image voxel, which enabled voxel‐vise current density calculation by fitting an appropriate SMD model curve to the measured SMD curve. Effect of the voxel size on the signal decay and precision of the current density calculation was studied as well.

**Results:**

It was shown theoretically, as well as verified by experiments on test and biological samples, that the current flowing though the sample creates an inhomogeneous magnetic field, which, as a consequence has a faster signal decay. Estimated current density from the measured signal decay increase agreed reasonably well with the actual current density, especially with the larger voxel sizes and longer times to signal acquisition. The sensitivity of the SMD method is up to $1/\sqrt{6}$ the sensitivity of the current density imaging method.

**Conclusion:**

SMD method of current detection is not limited to any particular sample orientation or geometry, and any pulse sequence capable of acquisition of the current‐induced signal evolution in a voxel can be used for it. This widens the scope of its application from tissues to in vivo studies on animals and humans.

## 1 INTRODUCTION

MR also enables imaging of the electric currents.^1^, ^2^ The current density imaging (CDI)^3^, ^4^ method is based on the application of the electric current in pulses that result in the precession phase shift. This is proportional to the duration of electric pulses and *z*‐component of the magnetic field change induced by the current. Firstly, this allows the calculation of the magnetic field change from the measured phase shift. Secondly, this allows the calculation of current density using Ampere's law from the magnetic field change. Use of the spin echo in CDI increases its sensitivity^5^ and thus enables the CDI microscopy.^6^ A multi spin‐echo version of CDI also exists in which each refocusing RF pulse is succeeded by an electric pulse having an opposite polarity resulting in an alternating train of electric pulses (alternating current‐CDI sequence).^7^ Alternating currents can also be imaged in the kHz range based on a resonant interaction between an applied RF field and an oscillating magnetic field induced by the currents^8^ or at Larmor frequency using the RF‐CDI sequence.^9^ There were also attempts to image currents based on the Lorentz force effect.^10^

Applications of the electric current imaging range from biology and medicine, such as electrostimulation of brain,^11^ monitoring of defibrillation pulses,^12^ and electroporation treatment,^13^ to its utility in material research and technology, such as study of electroosmotic flow^14^ and transport properties in porous materials.^15^ Information on the current distribution in a sample for at least 2 nonequivalent current injection arrangements can be used to map the electrical impedance of the sample. This method is known as MR electrical impedance tomography.^16^, ^17^ One of the very challenging areas in the field of current detection by MRI is also the imaging of neuronal currents. So far, various approaches for the detection, ranging from frequency and phase shift effects to the Lorentz force effect, were analyzed mainly theoretically.^18^, ^19^, ^20^

Here an alternative method to CDI is presented, which is considerably simpler to implement, is still sensitive, and has sufficient spatial resolution. This method is based on the detection of the decrease in the magnetic field homogeneity due to electric currents in the sample.

## 2 METHODS

### 2.1 Principle of current detection by a signal decay increase

**FIGURE 1:**
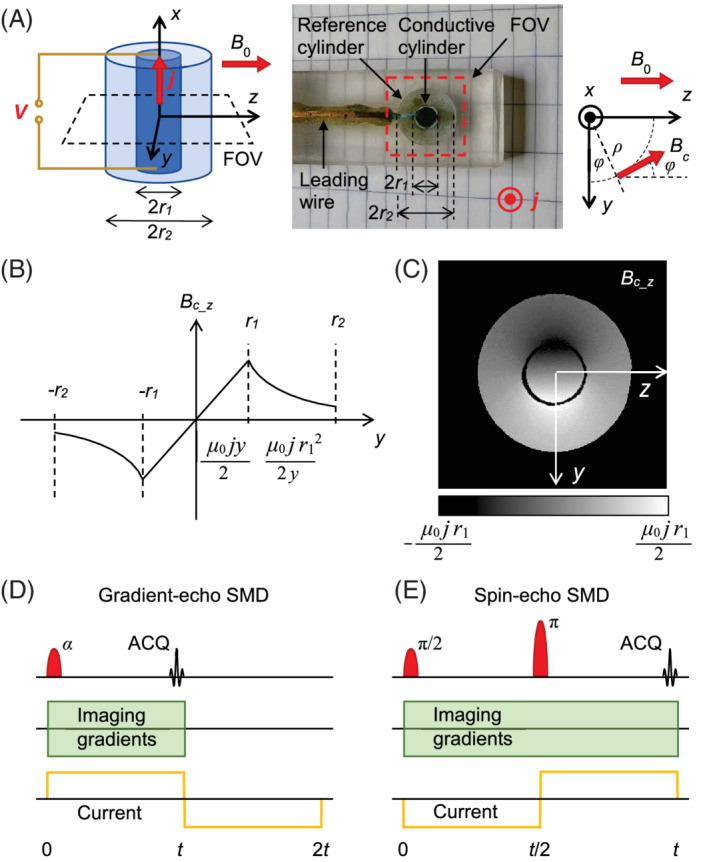
The sketch and photo of the sample (A) that was used for testing in the proposed signal decay current detection method. The sample consisted of 2 concentric cylindrical containers with the diameters of 4 mm and 10 mm, both which were filled with 2% saline. Only the inner cylinder was connected by the electrodes to the voltage supply of 10 V, and it was conducting current of 28 mA during the electric pulses. Axis of the cylinders were perpendicular to the direction of the static magnetic field *B*_0_ to maximize the *z*‐component of the magnetic field change $B_{c_{z}}$. Dependence of $B_{c_{z}}$ along the *y*‐axis (B) and its profile in the *yz*‐plane (C). It can be seen that $B_{c_{z}}$ increases linearly in the inner cylinder and decreases proportionally with the reciprocal radial distance from the cylinder axis in the outer cylinder. The scheme of gradient‐echo (D) and of spin‐echo (E) sequences with superimposed bipolar electric pulses that enables current detection by the signal magnitude decay (SMD).

Suppose that in a long cylindrically shaped volume with radius $r_{2}$, the electric current density $\vec{j}$ flows along the cylinder for radial distances $\rho \leq r_{1}$ and no current flows for larger distances $r_{1}<\rho \leq r_{2}$ (Figure 1A). Here, the radial vector is perpendicular to the current direction $\vec{\rho }=\vec{r}-\left(\vec{r}\cdot {\vec{e}}_{j}\right){\vec{e}}_{j}$; the radial distance is its magnitude $\rho =\left|\vec{\rho }\right|$; and ${\vec{e}}_{j}=\vec{j}/\left|\vec{j}\right|$ is the unit vector in the direction of the current. As follows from Ampere's law, this current creates a magnetic field ${\vec{B}}_{c}$ that is tangential to the cylinder and increases proportionally to ρ in the inner part of the cylinder with current and decreases as 1/ρ in the outer part of the cylinder without current (Figure 1B, 1C),

(1) $${\vec{B}}_{c}=\left\{\begin{array}{cc} \frac{\mu_{0}\;\vec{j}\times \vec{\rho }}{2}, & \rho \leq r_{1}\\ \frac{\mu_{0}{\;r}_{1}^{2}\;\vec{j}\times \vec{\rho }}{2\rho^{2}}, & \rho >r_{1} \end{array}\right..$$

Here, $\mu_{0}$ is the vacuum permeability. In order to affect the precession of the nuclei, the current must produce magnetic field component $B_{c_{z}}$ along the direction of the static magnetic field $B_{0}$. This magnetic field component is equal to

(2) $$B_{c_{z}}={\vec{B}}_{c}\cdot {\vec{e}}_{z}=\left\{\begin{array}{cc} \frac{\mu_{0}\;\vec{\rho }\cdot {\vec{j}}_{⊥}}{2}, & \rho \leq r_{1}\\ \frac{\mu_{0}{\;r}_{1}^{2}\;\vec{\rho }\cdot {\vec{j}}_{⊥}}{2\rho^{2}}, & \rho >r_{1} \end{array}\right..$$

Here, the relation $\left(\vec{j}\times \vec{\rho }\right)\cdot {\vec{e}}_{z}=\vec{\rho }\cdot \left({\vec{e}}_{z}\times \vec{j}\right)$ was used, where ${\vec{e}}_{z}$ is the unit vector in the *z*‐direction and ${\vec{j}}_{⊥}={\vec{e}}_{z}\times \vec{j}=\left(-j_{y},j_{x},0\right)$ so that ${\vec{j}}_{⊥}$ is perpendicular to the static magnetic field and current, that is, ${\vec{j}}_{⊥}\cdot {\vec{e}}_{z}=0$ and ${\vec{j}}_{⊥}\cdot {\vec{e}}_{j}=0$. The corresponding gradient of the magnetic field $B_{c_{z}}$ (Equation 2) is equal to

(3) $${\vec{G}}_{c}=\nabla B_{c_{z}}=\left\{\begin{array}{cc} \frac{\mu_{0}\;{\vec{j}}_{⊥}}{2}, & \rho \leq r_{1}\\ \frac{\mu_{0}{\;r}_{1}^{2}}{2}\left(\frac{{\vec{j}}_{⊥}}{\rho^{2}}-\frac{2\left(\vec{\rho }\cdot {\vec{j}}_{⊥}\right)\vec{\rho }}{\rho^{4}}\right), & \rho >r_{1} \end{array}\right.,$$

and its magnitude is

(4) $$G_{c}=\left|\nabla B_{c_{z}}\right|=\left\{\begin{array}{cc} \frac{\mu_{0}\;j_{⊥}}{2}, & \rho \leq r_{1}\\ \frac{\mu_{0}{\;r}_{1}^{2}\;j_{⊥}}{2\rho^{2}}, & \rho >r_{1} \end{array}\right.,$$

where $j_{⊥}=\left|{\vec{j}}_{⊥}\right|=\sqrt{j_{x}^{2}+j_{y}^{2}}$ denotes the magnitude of the current density component perpendicular to $B_{0}$. As seen from the Equations 3,4, the gradient of $B_{c_{z}}$ is proportional to the current density component perpendicular to $B_{0}$, whereas its direction is perpendicular to $\vec{j}$ and ${\vec{B}}_{0}$. Presence of the magnetic field gradient in the sample reshapes the FID signal and makes it decay faster. Let us examine this effect in a cubic voxel with a side of length L. In this voxel, magnetic field is also inhomogeneous to some extent when there is no current flowing through the voxel such that its signal then decays with the rate $1/T_{2}^{*}$

(5) $$S\left(t\right)=S_{0}\exp\left(-t/T_{2}^{*}\right).$$

During the application of current, the magnetic field gradient $G_{c}$ (Equation 4) is established in the voxel. A signal magnitude from the voxel in the presence of the magnetic field gradient $G_{c}$ can be calculated using the following equation

(6) $$\begin{array}{cc} S_{c}\left(t\right) & =S_{0}\exp\left(-t/T_{2}^{*}\right)\;\frac{1}{L}\left|\int_{-\frac{L}{2}}^{\frac{L}{2}}\exp\left(i\gamma G_{c}\mathrm{lt}\right)\mathrm{dl}\right|\\ & =S_{0}\exp\left(-t/T_{2}^{*}\right)\;\frac{\left|\sin\left(\gamma G_{c}\mathrm{Lt}/2\right)\right|}{\gamma G_{c}\mathrm{Lt}/2}. \end{array}$$

Normalization of the signal $S_{c}\left(t\right)$ (Equation 6) to the FID signal S(t) (Equation 5) and substitution of $G_{c}$ with the expression in Equation 4 for the inner cylinder with the homogeneous current yield a model for the normalized relaxation‐compensated signal magnitude decay (SMD) from the voxel

(7) $$\begin{array}{cc} f\left(t\right) & =\frac{S_{c}\left(t\right)}{S\left(t\right)}=\frac{\left|\sin\left(\gamma \mu_{0}j_{⊥}\mathrm{Lt}/4\right)\right|}{\gamma \mu_{0}j_{⊥}\mathrm{Lt}/4}\\ & =\frac{\left|\sin\left(\mathrm{Ct}\right)\right|}{\mathrm{Ct}},\;C=\frac{\gamma \mu_{0}j_{⊥}L}{4}. \end{array}$$

Determination of parameter C, for example, by fitting the model function in Equation 7 to experimental data for the normalized signal $S_{c}/S$, enables the calculation of current density

(8) $$j_{⊥}=\frac{4C}{\gamma \mu_{0}L}.$$

For small arguments Ct (short time t, low current density $j_{⊥}$, small voxel size L, or a combination of these), Equation 7 simplifies to

(9) $$f\left(t\right)\approx 1-\frac{{\left(\mathrm{Ct}\right)}^{2}}{6}.$$

Equation 8 then enables a simple estimation of current density $j_{⊥}$ from the measured normalized voxel signal

(10) $$j_{⊥}\approx \frac{\sqrt{96\left(1-f\left(t\right)\right)}}{\gamma \mu_{0}\mathrm{Lt}}.$$

Because both S(t) and $S_{c}\left(t\right)$ have random noise σ, the estimated current density has noise equal to

(11) $$\sigma_{j_{⊥}}\approx \sqrt{\frac{24\;\left(1+f^{2}\left(t\right)\right)}{1-f\left(t\right)}}\;\frac{1}{\gamma \mu_{0}\mathrm{Lt}\;\mathrm{SNR}\left(t\right)},$$

where SNR(t)=S(t)/σ is the SNR in the voxel without current at time t.

### 2.2 Samples

Experiments were performed on a test sample and biological sample *ex vivo*. Test sample (Figure 1A) consisted of 2 electrically isolated concentric cylindrical containers of 13 mm length and 4 mm and 10 mm diameters of the inner and outer container, respectively.^6^, ^7^ Both containers were filled with 2% saline, whereas only the inner container was capped with copper electrodes, conducting current of 28 mA (current density of 2200 A/m^2^) during the electric pulses. The outer cylinder was used as a zero‐current reference. The cylinder axis (*x*‐axis) was perpendicular to the static magnetic field of the magnet (*z*‐axis). Biological sample was a fresh lower chicken thigh in which 1 mm diameter platinum–iridium needle electrodes spaced by 11 mm and oriented along the static magnetic field were inserted. The electrodes delivered bipolar electric pulses of 30 V that resulted in the current of amplitude 40 mA.

### 2.3 Imaging of current distribution by the SMD method

**FIGURE 2:**
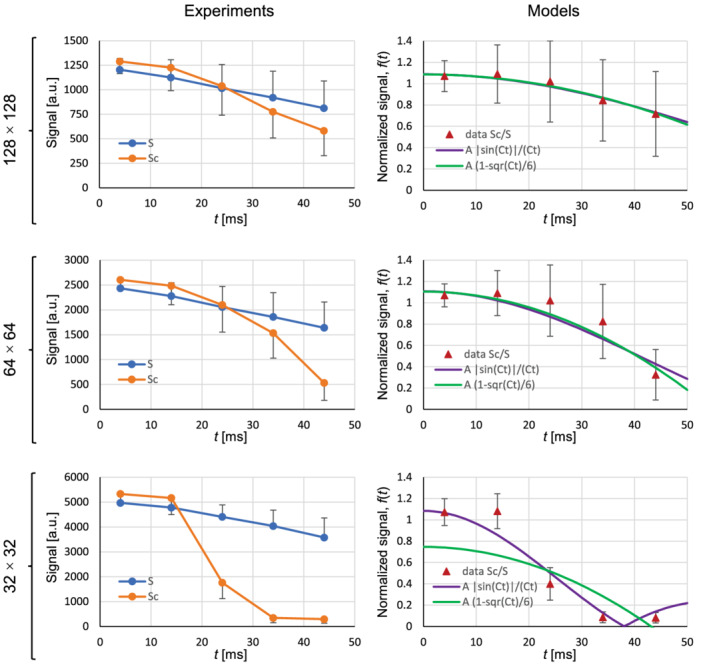
Graphs in the left column show the average signal magnitude from the voxel in the inner cylinder as a function of time *t* (from signal excitation to signal acquisition) for different imaging matrix sizes (voxel sizes *L* = 117, 234, 469 μm). Signals *S*_*c*_ (orange curve) and *S* (blue curve) correspond to the case with and without current flowing through the inner cylinder, respectively. The graphs in the right column show the normalized signals *f*(*t*) (ratios between signals *S*_*c*_ and *S*) as a function of time *t* by experimental points (red triangles), and the best fit model curves for the signal magnitude decay model in Equation 7 (violet curve) and for its simplification in Equation 9 (green curve). All signals for the graphs were obtained from the images in Supporting Information Figure S2

The test sample was scanned with a FLASH‐type of gradient‐echo imaging sequence^21^, ^22^ (Figure 1D) using parameters: FOV 15 mm; TE 4, 14, 24, 34, 44 ms; TR 100 ms; and number of averages 2. Sample scanning was repeated for 3 different imaging matrices (voxel dimensions): 128 × 128 (117 × 1117 × 4000 μm^3^),64 × 64 (234 × 234 × 4000 μm^3^), and 32 × 32 (469 × 469 × 4000 μm^3^). Current was constant during t=TE period, and then its direction was reversed for the equal period to mitigate electrolysis of electrolyte. Due to the shorter $T_{2}^{*}$ relaxation time, lower conductivity, and therefore a need of longer current injection times t=TE for the biological sample, this sample was scanned with the modified spin‐echo imaging sequence (Figure 1E) using parameters: FOV 30 mm; imaging matrix (voxel dimension) 64 × 64 (469 × 469 × 4000 μm^3^); TE 20, 40, 60 ms; TR 1000 ms; and number of averages 8. In this sequence, simultaneous current and “time” reversals result in an undisturbed signal evolution due to currents and canceled effects of static magnetic field inhomogeneities on the signal at t=TE. This signal is effectively equal to that of the gradient‐echo sequence in a perfectly homogeneous magnetic field where $T_{2}^{*}=T_{2}$ (Supporting Information Figure S1). Both samples were scanned in a single 4 mm thick transverse slice (Figure 1A) and 2 different sample states: with and without current. All the experiments were performed on an MRI system consisting of a 2.35 Tesla horizontal bore superconducting magnet (Oxford Instruments, Abingdon, UK), an Apollo NMR/MRI spectrometer (Tecmag, Houston TX, USA), and accessories for micro imaging (Bruker, Ettlingen, Germany).

## 3 RESULTS

The graphs in the left column of Figure 2 show time dependence of the average voxel signal magnitudes S (blue curve) and $S_{c}$ (orange curve) in the inner cylinder of the test sample without and with the current, respectively. The signals were measured from signal magnitude images of the test sample in Supporting Information Figure S2 for all the 3 different voxel sizes L (imaging matrices). These graphs confirm the trend, which was observed in the gradient‐echo images of the test sample; namely, that the signal decay of the test sample with current is faster than that of the sample without the current and also that the decay is faster with the larger voxel sizes L. This trend is reflected in the signal phase images in Supporting information Figure S3 with faster changing phase. Each signal $S_{c}$ was then divided by the corresponding signal S to obtain experimental normalized signals $S_{c}/S$. These signals are shown by red triangles, along with the best‐fit model curves in graphs in the right column of Figure 2. Violet model curves correspond to the SMD model given by the Equation 7, and green curves correspond to the simplified SMD model given by the Equation 9. It can be seen that both models produce approximately equivalent results for low and medium signal decays (L = 117, 234 μm; matrix 128 × 128, 32 × 32), whereas the simplified model fails with the higher signal decay (L = 469 μm; matrix 32 × 32).

In Table 1, results of the multi‐ and single‐point analysis of the $S_{c}/S$ data from Figure 2 are shown. It can be seen that from the multi‐point analysis, that is, fitting of the SMD model to the $S_{c}/S$ data, both SMD models yielded practically identical model parameters C and relatively high coefficients of determination *R* ^2^ for the smaller and medium voxel sizes (L = 117, 234 μm), whereas the simplified model yielded too low model parameter C and also low coefficient of determination of *R* ^2^ = 0.49 for the larger voxel size (L = 469 μm). Corresponding current densities $j_{⊥}$, which were calculated from parameters C using the Equation 8, were in the range from 1400 to 3400 A/m^2^, whereas the actual value was 2200 A/m^2^. The most accurate results for $j_{⊥}$ were obtained for the medium voxel size (L = 234 μm) with both models, and for the larger voxel size (L = 469 μm) with the model in Equation 7. The simplified model (Equation 9) yielded overestimated $j_{⊥}$ with the smaller voxel size (L = 117 μm) and underestimated $j_{⊥}$ with the larger voxel size (L = 469 μm). The simplified model was also used for the calculation of the current density $j_{⊥}$ from a single data point using the Equation 10; that is, from a normalized signal $S_{c}/S$ at t = 34 or t = 44 ms. Calculated $j_{⊥}$ was in the range from 1400 to 2900 A/m^2^, which is comparable with the corresponding results obtained by the multi‐point analysis. However, the errors of $j_{⊥}$ (Equation 11) were considerably higher with this method, especially for the smaller voxel size L = 117 μm.

**TABLE 1:** Estimated current density $j_{⊥}$ in the inner cylinder of the test sample. In multi‐point analysis, parameters A and C of model function f(t)=A|sin(Ct)|/(Ct) (Equation 7) and its simplification $f\left(t\right)=A\left(1-{\left(\mathrm{Ct}\right)}^{2}/6\right)$ (Equation 9) were obtained by the best fit of the model to all the normalized signals $S_{c}/S$, whereas in single‐point analysis, parameter C and its error were calculated from a normalized signal $S_{c}/S$ at t = 34 ms and t = 44 ms using the simplified model function with *A* = 1 (Equations 10,11). The parameter C was then used to calculate the estimate for current density $j_{⊥}$ using Equation 8. The normalized signal $S_{c}/S$ was determined from average signals $S_{c}$ and S in the inner cylinder region (Supporting Information Figure S2).

Model *f*(*t*)		*L* [μm]	*A*	*σ*_*A*_	*C* [s^−1^]	*σ*_*C*_ [s^−1^]	*χ* ^2^	*R* ^2^	*j*_*┴*_ [A/m^2^]	*σ*_*j*_ [A/m^2^]
Multi‐point	$A\frac{\left|\sin\left(\mathrm{Ct}\right)\right|}{\mathrm{Ct}}$	117	1.088	0.018	33.8	2.5	0.02	0.93	3425	251
		234	1.107	0.051	49.1	5.2	0.27	0.88	2491	265
		469	1.084	0.118	82.5	3.5	1.45	0.94	2093	89
	$A\left(1-\frac{C^{2}t^{2}}{6}\right)$	117	1.086	0.016	32.3	2.0	0.02	0.94	3274	202
		234	1.105	0.042	44.8	3.0	0.18	0.92	2270	152
		469	0.747	0.270	56.7	5.5	11.39	0.49	1439	138
Single‐point	$1-\frac{C^{2}t^{2}}{6}$ *t* = 34 ms	117			28.6	34.8			2898	3528
		234			30.2	29.5			1528	1494
		469			69.1	6.0			1747	152
	$1-\frac{C^{2}t^{2}}{6}$ *t* = 44 ms	117			29.7	28.4			3010	2876
		234			45.8	14.5			2320	736
		469			53.5	8.3			1353	210

**FIGURE 3:**
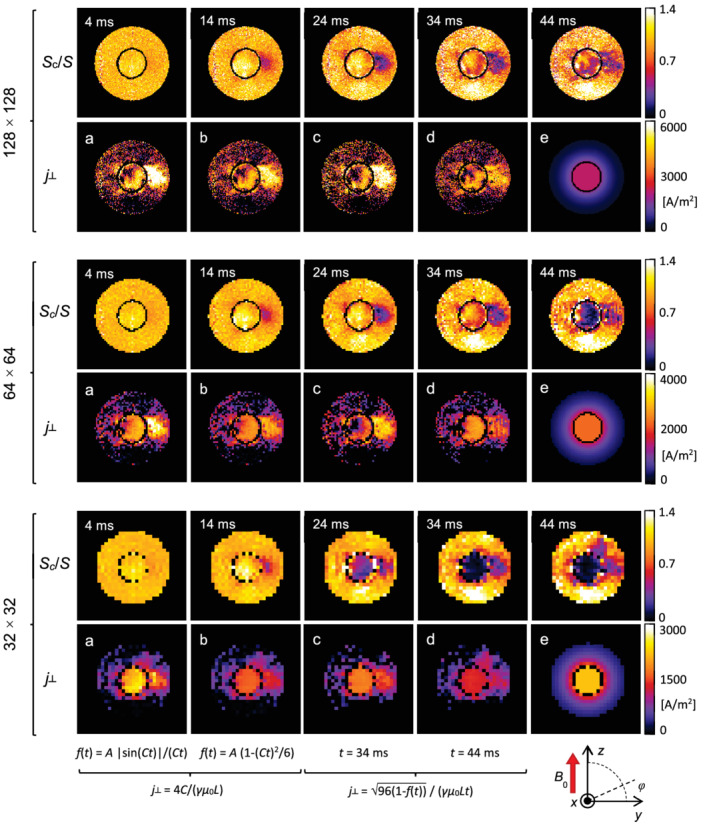
Images in the upper rows show measured normalized signals $S_{c}/S$ that were obtained by dividing “current” with the corresponding “no‐current” magnitude images from Supporting Information Figure S2, acquired by the gradient‐echo sequence in Figure 1D. Images of the normalized signals are shown for different current injection times *t* and voxel sizes *L* = 117, 234, and 469 μm (imaging matrices 128 × 128, 64 × 64, and 32 × 32). Images in the lower rows show current density $j_{⊥}$ calculated pixel‐wise using 4 different ways. By multi‐point analyses: a) model function f(t)=A|sin(Ct)|/(Ct) or b) simplified model function $f\left(t\right)=A\left(1-{\left(\mathrm{Ct}\right)}^{2}/6\right)$ was first fitted to $S_{c}/S$ data to obtain the parameter *C*, which was then utilized to calculate the corresponding current density $j_{⊥}$ using Equation 8. In single‐point analyses, current density $j_{⊥}$ was calculated from a single normalized signal value using the simplified model in Equation 10 for 2 different times: c) *t* = 34 ms and d) *t* = 44 ms. Maps e) show theoretically expected current density $j_{⊥}$ that was calculated from magnetic field gradient given in Equation 4 for the inner cylinder

In the upper rows of Figure 3 are shown images of the normalized signals $S_{c}/S$ obtained by pixel‐wise division of “current” with the corresponding “no‐current” test sample magnitude images from Supporting Information Figure S2. These images were used to calculate images of the current density $j_{⊥}$, shown in Figure 3 (lower rows). These were calculated by the same 4 methods that were used for the calculation of $j_{⊥}$ in Table 1; however, in a pixel‐wise manner. It can be seen that the most accurate results for $j_{⊥}$ image were obtained with the model in Equation 7 using medium and larger voxel size (L = 234, 469 μm, matrix 64 × 64, 32 × 32). With these voxel sizes and the simplified model, either multi‐point (Equation 9) or single‐point (Equation 10), intermediate quality results were acquired, whereas poor quality results were obtained with the smaller voxel size (L = 117 μm, matrix 128 × 128).

**FIGURE 4:**
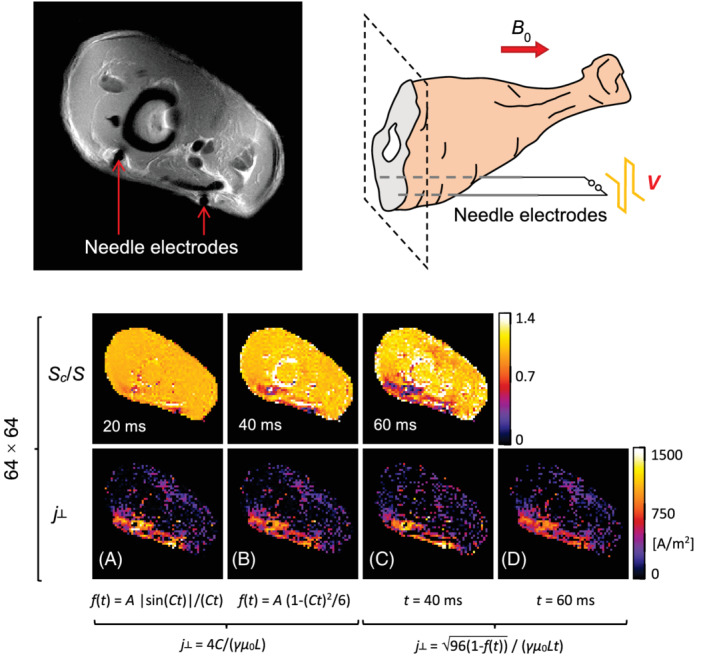
The current density imaging by the SMD method on the biological (lower chicken thigh) sample *ex vivo*. The sample was imaged in a single transversal 4 mm thick slice perpendicular to the static magnetic field and to the needle electrodes spaced by 11 mm; bipolar electric pulses of 30 V and 40 mA were delivered to the electrodes. Position of the electrodes in the sample is shown in the grayscale image. Images in the upper row show measured normalized signals $S_{c}/S$ that were obtained by dividing “current” with the corresponding “no‐current” magnitude images from Supporting Information Figure S4 acquired by the spin‐echo sequence in Figure 1E. These images are shown for 3 different current injection times *t* and voxel size *L* = 469 μm (imaging matrix 64 × 64). Images in the lower row show current density $j_{⊥}$ calculated pixel‐wise using the same 4 different methods utilizing for the test sample in Figure 3, that is, by using: (a) model function f(t)=A|sin(Ct)|/(Ct), (b) simplified model function $f\left(t\right)=A\left(1-{\left(\mathrm{Ct}\right)}^{2}/6\right)$, (c) single‐point analysis with *t* = 40 ms, and (d) single‐point analysis with *t* = 60 ms

By using the spin‐echo sequence on the biological (chicken lower thigh) sample ex vivo, similar quality results to those on the test sample were obtained despite more difficult conditions (lower conductivity and shorter $T_{2}^{*}$). These are shown in Figure 4 by images of the normalized signals $S_{c}/S$ (upper row) and the corresponding images of the current density $j_{⊥}$ (lower row), which were calculated from these with the same 4 methods used for the test sample in Figure 3. The normalized signal images were obtained the by pixel‐wise division of “current” with the corresponding “no‐current” biological sample magnitude images from Supporting Information Figure S4. These images have low noise and artifacts, which enabled clearly visible regions with a reduced signal due to current. These regions coincide with the regions of higher current density in the calculated $j_{⊥}$ images. Best results among these were obtained with the model a) (Equation 7) and the single‐point model c) with *t* = 40 ms.

## 4 DISCUSSION

An effect of the large current‐time products on the signal loss in MR magnitude images was reported already in 1992.^5^ However, to the best of author's knowledge, this effect, which is essentially $T_{2}^{*}$ effect in the proposed SMD method, has not been used thus far as a current detection method. The SMD method senses magnitude of current‐induced magnetic field gradient and uses it for the estimation of $j_{⊥}$. SMD has lower spatial resolution than CDI; however, its advantage over CDI is in simplicity. It can be performed in a single sample orientation by any sequence capable of detecting current‐induced signal magnitude decay in a voxel. On the other hand, conventional CDI is associated with reorienting the sample in the magnet to all 3 mutually perpendicular orientations to measure all 3 components of the magnetic field change ${\vec{B}}_{c}$, which is often difficult or even impossible to perform. For this reason, there were several attempts to perform CDI only in a single sample orientation, for example, by the projected current density method.^23^ All these are associated with the larger errors that can usually be mitigated by complex further processing of the measured data with numerical modeling based on the actual geometry of the sample and known electric potentials.

The proposed SMD method associates regions having a magnetic field gradient with the current density based on the relation $j_{⊥}=2G_{c}/\mu_{0}$. Because this relation was derived for the cylindrical region with the homogeneous current distribution, this method, in general, provides only an approximate solution for current density in regions of other shape and current distribution. For example, for the outer cylinder region of the test sample where the magnetic field gradient is still present (Equation 4), this method yields $j_{⊥}=I/\left(\pi \rho^{2}\right)$ instead of $j_{⊥}=0$; here, I is current in the inner cylinder. Results on the test sample show that current density estimation is erroneous in transition between 2 regions of different current density, and this error decays proportionally to an inverse radial distance squared from the region's center. This error can also be well seen experimentally in images of $j_{⊥}$ in Figure 3. In addition, the gradient‐echo SMD method (Equation 6) assumes that the effects of the static magnetic field gradient $\nabla B_{0}$ and the current‐induced magnetic field gradient ${\vec{G}}_{c}$ on the signal decay are mutually independent. However, this is only a simplification; it does not take into consideration the possible effect of interference between these 2 gradients (mixed term ${\vec{G}}_{c}\cdot \nabla B_{0}$) on the signal. The presence of this interference also possibly explains the local differences between the experimental and simulated images of $j_{⊥}$ in Figure 3.

From Equation 11 for the current noise $\sigma_{j_{⊥}}$, it is evident that the noise is inversely proportional to the voxel size L and the time‐SNR product tSNR(t). This equation is also very similar to the equation for the current noise in the conventional CDI experiment^5^, ^6^

(12) $$\sigma_{j}=\frac{2}{\gamma \mu_{0}LT_{c}\;\mathrm{SNR}}.$$

Here, L is the CDI voxel size; $T_{c}$ is the current injection time; and SNR relates to the magnitude image. In the CDI method, signal is acquired using the spin‐echo imaging sequence, and $T_{c}$ can practically extend over all t=TE period so that the sensitivity of CDI experiment, which is defined as $1/\sigma_{j}$, is proportional to $t\;\exp\left(-t/T_{2}\right)$ and $LV_{1}$, where $V_{1}$ is the voxel volume. In the proposed SMD method, signal is acquired using the gradient‐echo or spin‐echo imaging sequence so that its sensitivity is proportional to $t\;\exp\left(-t/T_{2}^{*}\right)$ or $t\;\exp\left(-t/T_{2}\right)$ multiplied by $\sqrt{1-f\left(t\right)}/\sqrt{1+f^{2}\left(t\right)}$, and it is also proportional to $LV_{1}$ according to Equation 11. Thus, the ratio between sensitivities of the CDI and the SMD method is equal to

(13) $$\begin{array}{cc} & \frac{{\text{sensitivity}}_{\mathrm{CDI}}}{{\text{sensitivity}}_{\mathrm{SMD}}}=\sqrt{\frac{6\left(1+f^{2}\left(t\right)\right)}{1-f\left(t\right)}}\\ & \cdot \left\{\begin{array}{cc} \exp\left(\left(1/T_{2}^{*}-1/T_{2}\right)t\right), & \text{Gradient‐echo}\;\mathrm{SMD}\\ 1, & \text{Spin‐echo}\;\mathrm{SMD} \end{array}\right.. \end{array}$$

Because 0<f(t)<1 sensitivity of the CDI method is always at least a factor $\sqrt{6}$ higher than the sensitivity of the SMD method, this deficiency in the sensitivity of SMD method can be compensated by an increase in the voxel volume, that is, the factor $LV_{1}$. For cubic voxel with size L, where $V_{1}=L^{3},$ this factor is equal to $L^{4}$. Voxel size has, therefore, a big impact on the sensitivity. For example, doubling the voxel size L results in the 16‐fold increase in the sensitivity. SNR of the current density image ${\mathrm{SNR}}_{j}=j/\sigma_{j}$ can also be increased by increasing the applied current. If in the SMD method, this is increased by a factor equal to the ratio of sensitivities in Equation 13, and then ${\mathrm{SNR}}_{j}$ of the SMD method is equal to or higher than ${\mathrm{SNR}}_{j}$ of the CDI method.

As can be seen from Equation 13, the sensitivity of the spin‐echo SMD method is always higher than the sensitivity of the gradient‐echo SMD method, namely, $T_{2}^{*}\leq T_{2}$. This makes the spin‐echo SMD more appropriate for use with the samples in which longer current injection times are needed, for example, in all biomedical applications. However, advantage of gradient‐echo SMD is that it can be performed with constant currents, whereas in spin‐echo SMD bipolar currents synchronized with the imaging sequence are needed.

## 5 CONCLUSION

In this study, it is demonstrated that the electric currents can also be effectively detected by MR based on the increase in signal magnitude decay that is induced by the current. This approach is simpler than the conventional CDI method; all needed information for estimation of $j_{⊥}$ is acquired in just 1 sample orientation using any pulse sequence capable of detecting current‐induced signal magnitude decay in a voxel. This method can potentially be used in different fields of medicine where there is a need to monitor distribution of electric currents during a diagnostic or therapeutic procedure.

## Supporting information


**Figure S1.** Scheme of phase shift time evolution in gradient‐echo and spin‐echo sequences with superimposed electric current pulses. φ_s_ (red curve) corresponds to the phase shift due to static magnetic fields, while φ_c_ (yellow curve) corresponds to the phase shift due to magnetic fields created by the electric currents.
**Figure S2.** Magnitude images of the 4 mm thick central slice in the *yz* orientation across the test sample. The images were acquired with the gradient‐echo imaging sequence at different times *t* = 4, …44 ms after the signal excitation using imaging matrices 128×128, 64×64 and 32×32 in case of the current flowing through the inner cylinder of the test sample (lower rows) and without it (upper rows). All the images were acquired at the FOV of 15 mm so that voxel sizes (along *y*‐direction) were equal to *L* = 117, 234 and 469 μm. It can be seen that the signal decreases faster in case of the current flowing through the sample than without it. This decrease is faster with a larger voxel size and is especially apparent in the inner cylinder region.
**Figure S3.** Phase images that correspond to signal magnitude images in Figure S2. It can be seen that in the regions with a higher signal loss in the magnitude images, the phase gradient is higher. This is especially apparent in the inner cylinder region with longer current injection times *t*.
**Figure S4.** Signal magnitude (A) and the corresponding signal phase (B) images of the 4 mm thick central slice in the transversal orientation across the lower chicken thigh. Slice orientation was also perpendicular to the static magnetic field and the electrodes. The images were acquired with the spin‐echo imaging sequence using synchronized electric pulses (Figure 1E) at current injection times *t* = 20, 40, 60 ms (also equal to TE), imaging matrix 64×64, and in two states of the sample: with current (lower rows) and without it (upper rows). All the images were acquired at the FOV of 30 mm so that the voxel sizes (along *xy*‐direction) were equal to *L* = 469 μm. Images of the sample with current have significantly less signal in regions with a higher injected current (current‐time products), i.e., in proximity of the electrodes and the region between them where current density is higher and with longer times (*t* = 40, 60 ms). In phase images, these regions coincide with the regions of a higher phase gradient.
