# Supplementary material for: Electric Current Detection Based on the MR Signal Magnitude Decay
Source: Magn Reson Med. 2022 May 5;88(3):1282–91. doi: 10.1002/mrm.29278 (PMC9325414; doi:10.1002/mrm.29278)
Supplement: Supplementary file 1 — Figure S1. Scheme of phase shift time evolution in gradient‐echo and spin‐echo sequences with superimposed electric current pulses. φs (red curve) corresponds to the phase shift due to static magnetic fields, while φc (yellow curve) corresponds to the phase shift due to magnetic fields created by the electric currents. Figure S2. Magnitude images of the 4 mm thick central slice in the yz orientation across the test sample. The images were acquired with the gradient‐echo imaging sequence at different times t = 4, …44 ms after the signal excitation using imaging matrices 128×128, 64×64 and 32×32 in case of the current flowing through the inner cylinder of the test sample (lower rows) and without it (upper rows). All the images were acquired at the FOV of 15 mm so that voxel sizes (along y‐direction) were equal to L = 117, 234 and 469 μm. It can be seen that the signal decreases faster in case of the current flowing through the sample than without it. This decrease is faster with a larger voxel size and is especially apparent in the inner cylinder region. Figure S3. Phase images that correspond to signal magnitude images in Figure S2. It can be seen that in the regions with a higher signal loss in the magnitude images, the phase gradient is higher. This is especially apparent in the inner cylinder region with longer current injection times t. Figure S4. Signal magnitude (A) and the corresponding signal phase (B) images of the 4 mm thick central slice in the transversal orientation across the lower chicken thigh. Slice orientation was also perpendicular to the static magnetic field and the electrodes. The images were acquired with the spin‐echo imaging sequence using synchronized electric pulses (Figure 1E) at current injection times t = 20, 40, 60 ms (also equal to TE), imaging matrix 64×64, and in two states of the sample: with current (lower rows) and without it (upper rows). All the images were acquired at the FOV of 30 mm so that the voxel sizes (along xy‐direction [file MRM-88-1282-s001.pdf]

Supporting Information

Electric Current Detection Based on the MR Signal Magnitude Decay

Igor Serša [1,2]

[1]*Jožef Stefan Institute, Ljubljana, Slovenia*

[2]*Institute of Pathophysiology, Faculty of Medicine, University of Ljubljana, Ljubljana, Slovenia*

1 Electric pulses in spin-echo sequences

The spin-echo sequences are designed to cancel effects of all static magnetic fields on spin dephasing and thus allow formation of the spin-echo. This is enabled by the use of the 180° RF pulse that inverts the accumulated phase shift. Inversion of the phase shift on the echo signal is effectively equal to inversion of time after the 180° RF pulse. Suppose that the accumulated phase shift due to static magnetic fields prior to the 180° RF pulse is equal to α, then the phase shift is equal to $-\alpha$ immediately after the 180° RF pulse and is equal to $-\alpha + \alpha = 0$ in the spin-echo. As this result is independent of α, it is also independent of the static magnetic fields so that all spins have identical phase which leads to the spin-echo. However, in our case situation is more complex as the goal is to cancel the effect of all the static magnetic fields except those that have an origin in electric current. For this reason, electric currents in the spin-echo sequence (Figure 1E) change direction after the 180° RF pulse so that their magnetic fields change the sign from $-B_{c_z}$ to B_{c_z}. The accumulated phase shift before the 180° RF pulse $\alpha - \beta$ has now two contributions: α due to static magnetic fields and $-\beta = \gamma(-B_{c_z})t/2$ due to electric currents. Immediately after the 180° RF pulse, this phase inverts to $-\alpha + \beta$ and it increases by $\alpha + \beta$ till the spin-echo so that there it is equal to $-\alpha + \beta + \alpha + \beta = 2\beta$. This result confirms that this echo signal is equivalent to the one that would be obtained with the gradient-echo sequence in case of constant current and perfectly homogeneous static magnetic field ($\alpha = 0$). This is also depicted schematically in Figure S4 where phase shift time evolution due to static magnetic fields and due to magnetic fields created by electric currents in gradient-echo and spin-echo sequences is compared.

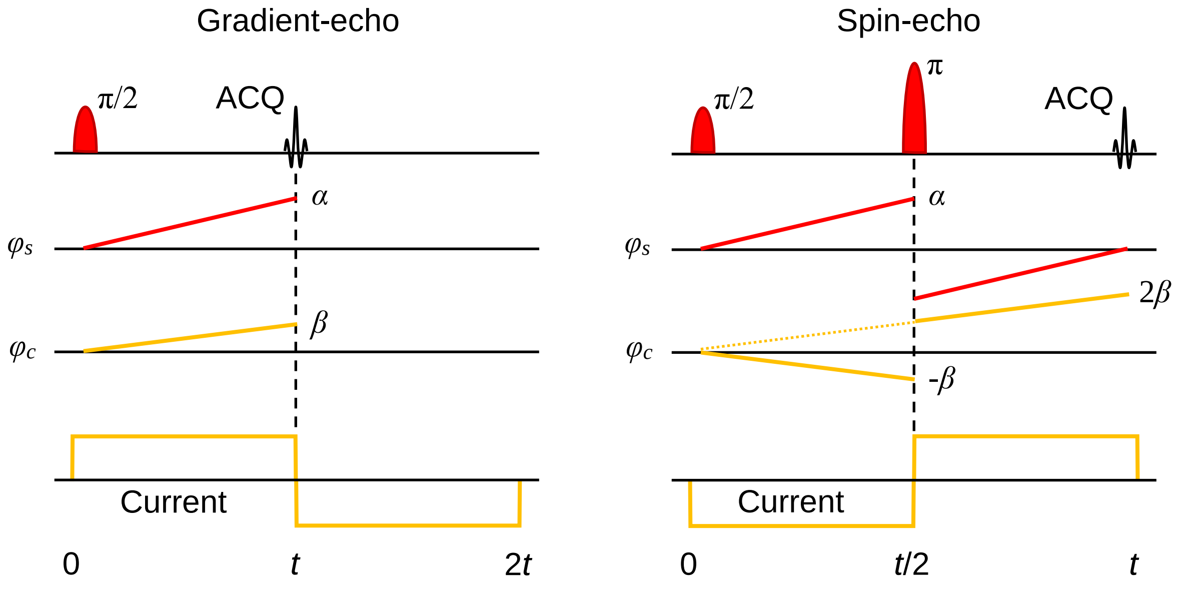

FIGURE S1. Scheme of phase shift time evolution in gradient-echo and spin-echo sequences with superimposed electric current pulses. φ_s (red curve) corresponds to the phase shift due to static magnetic fields, while φ_c (yellow curve) corresponds to the phase shift due to magnetic fields created by the electric currents.

2 Magnitude and phase images of the test and biological sample

Figures S2 and S3 show the magnitude and the corresponding phase images of the test sample in the central transverse (*yz*) slice with and without current flowing along the inner cylinder of the test sample. The images were acquired by the gradient-echo imaging sequence at different current injection times t = 4, 14, 24, 34, 44 ms (also equal to gradient-echo times TE, Figure 1D), repetition time TR =100 ms and for three different voxel sizes L = 117, 234, 469 μm that correspond to the imaging matrices 128×128, 64×64 and 32×32. Here, L is the voxel dimension along the y-direction, i.e., the direction perpendicular to B_0 and the current: This is also the direction of the B_{c_z} gradient in the inner cylinder. It can be seen that the signal (image intensity) decreases with an increasing time t in all signal magnitude images. The decrease is more intense in images that correspond to the sample with the current and also in the images with larger voxel sizes L. The signal loss is especially apparent in the inner cylinder, which is the region with the current and where the B_{c_z} gradient is the highest. From the signal phase images, it can be observed that the phase is most uniform with the shortest time t = 4 ms. With larger times t, this uniformity is lost progressively in both states: with current and without it. However, there is a distinct difference between these two states. In the phase images of the sample without current, there is no significant difference in phase uniformity between the inner and outer cylinder region, while in the phase images of the sample with current, phase uniformity in the inner cylinder region (where current flows) is significantly lower than in the outer cylinder region (where is no current). Phase in the inner cylinder has a constant gradient in the horizontal (y) direction. This is exactly what is expected according to the theory (Figure 1C). The phase gradient is higher with the higher current injection time. It can also be seen that the phase becomes random in regions where the magnitude signal decreases to the noise level or below (matrix 32×32, with current, t = 34, 44 ms).

In case of the biological sample (lower chicken thigh) in Figure S4, similar experiments were performed with the imaging parameters: current injection times t = 20, 40, 60 ms (also equal to the spin-echo time TE), repetition time TR = 1000 ms, voxel size L = 469 μm and imaging matrix 64×64. These images were acquired with the spin-echo imaging sequence using the bipolar electric pulses (Figure 1E). This was absolutely needed as the T_2^* was too short and sample conductivity was too low to enable quality images of the normalized signal S_c/S needed for the calculation of current density images. As seen in Figure S4A, these signal magnitude images have a good signal to noise ratio in both states of the sample: with and without current. However, a more careful inspection reveals that the images of the sample with current have less signal in regions close to the electrodes and between them than the corresponding images of the sample without current. This effect is more pronounced with the higher current injections (higher current-time products). Effect of current can be observed better in the signal phase images shown in Figure S4B. The phase images of the sample without current are very uniform with all current injection times t, i.e., considerably more than phase images of the test sample without current shown in Figure S3. This is due to the perfectly canceled effects of all the static magnetic field inhomogeneities in the spin-echo images. Phase images of the biological sample with current have a distinct pattern of changing phase from 0 (dark) to 2π (bright), which is very intense (phase gradient is high) with higher current injections in proximity of the electrodes and the region between them.

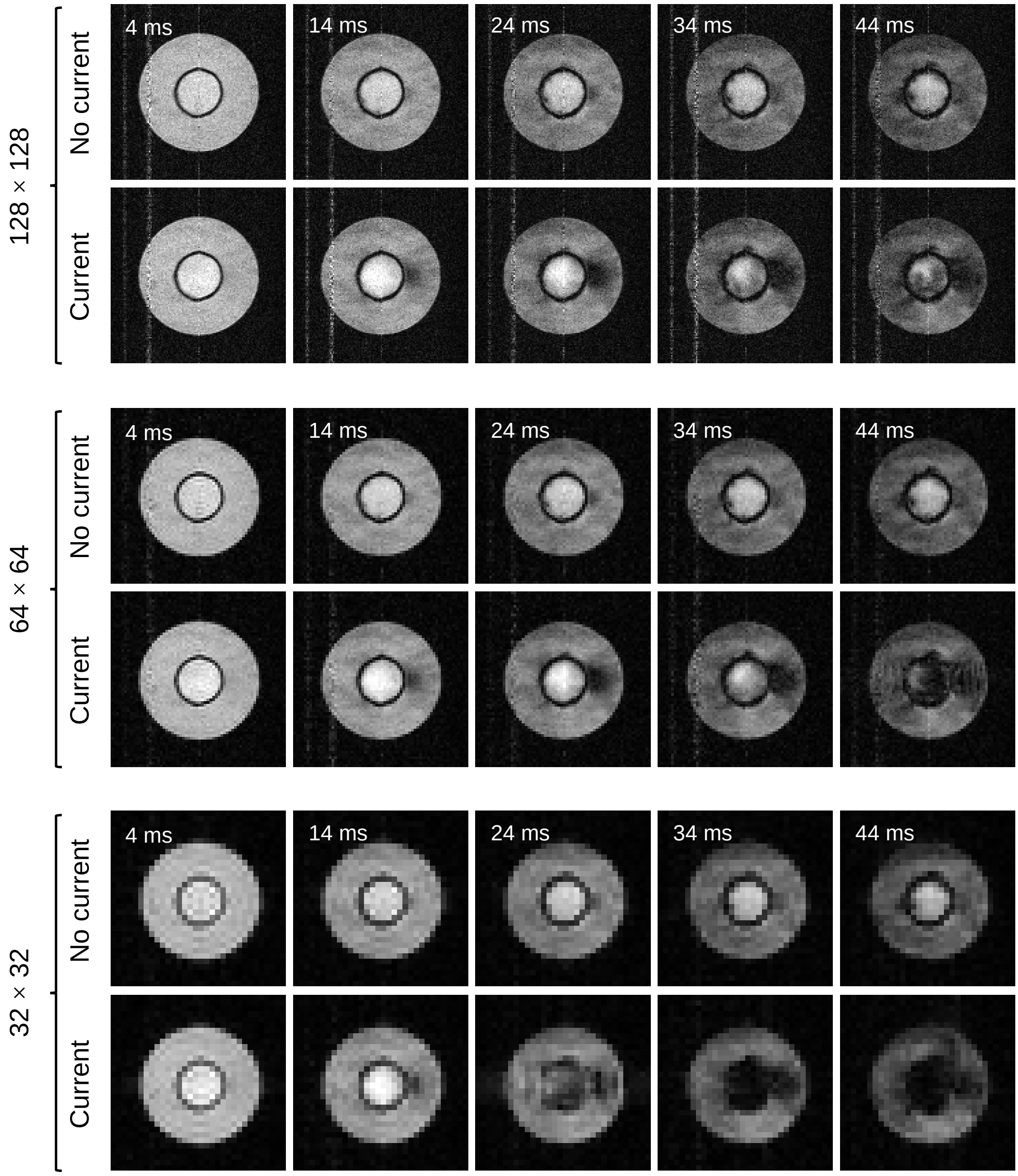

FIGURE S2. Magnitude images of the 4 mm thick central slice in the *yz* orientation across the test sample. The images were acquired with the gradient-echo imaging sequence at different times t = 4, …44 ms after the signal excitation using imaging matrices 128×128, 64×64 and 32×32 in case of the current flowing through the inner cylinder of the test sample (lower rows) and without it (upper rows). All the images were acquired at the FOV of 15 mm so that voxel sizes (along y-direction) were equal to L = 117, 234 and 469 μm. It can be seen that the signal decreases faster in case of the current flowing through the sample than without it. This decrease is faster with a larger voxel size and is especially apparent in the inner cylinder region.

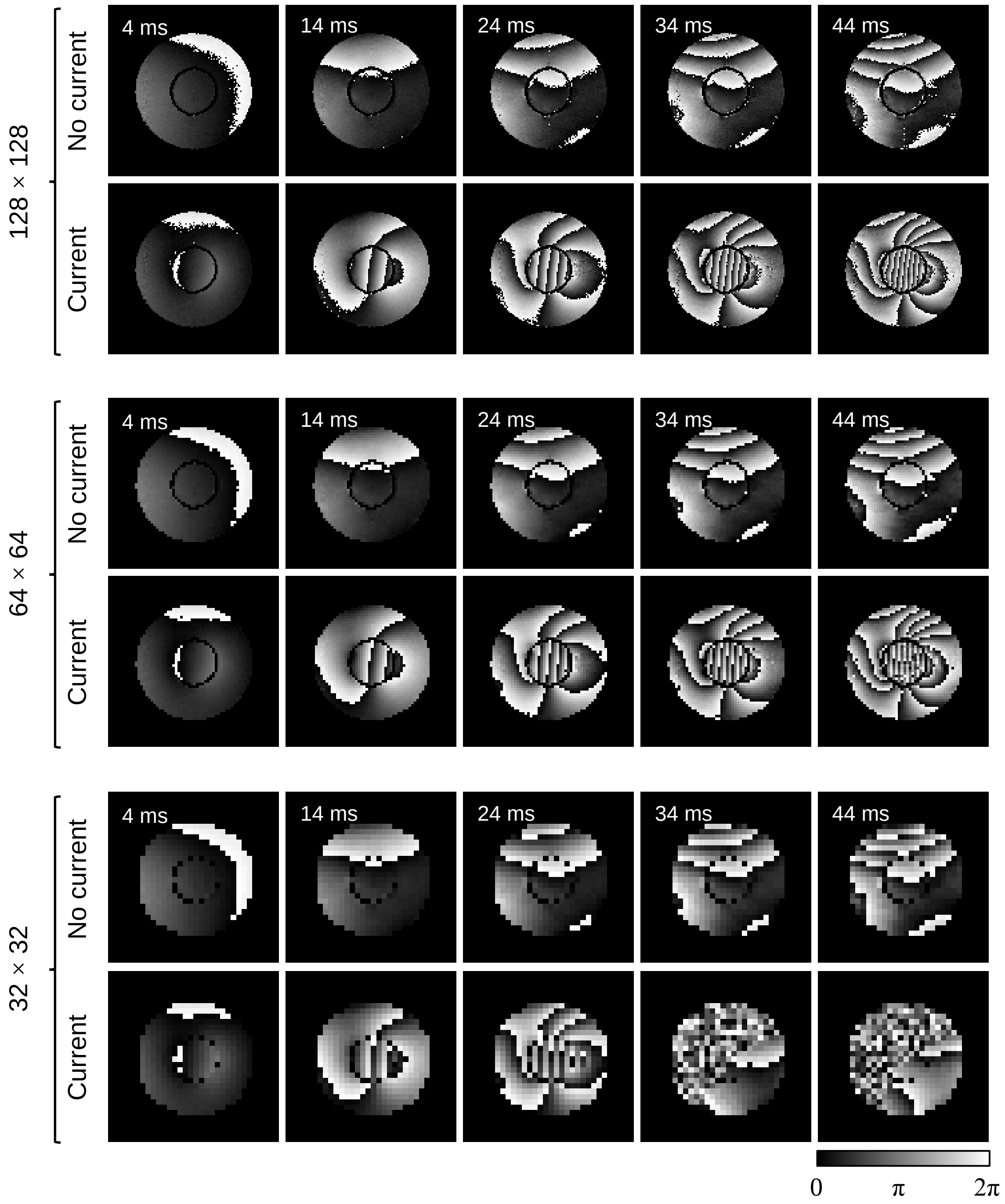

FIGURE S3. Phase images that correspond to signal magnitude images in Figure S2. It can be seen that in the regions with a higher signal loss in the magnitude images, the phase gradient is higher. This is especially apparent in the inner cylinder region with longer current injection times *t*.

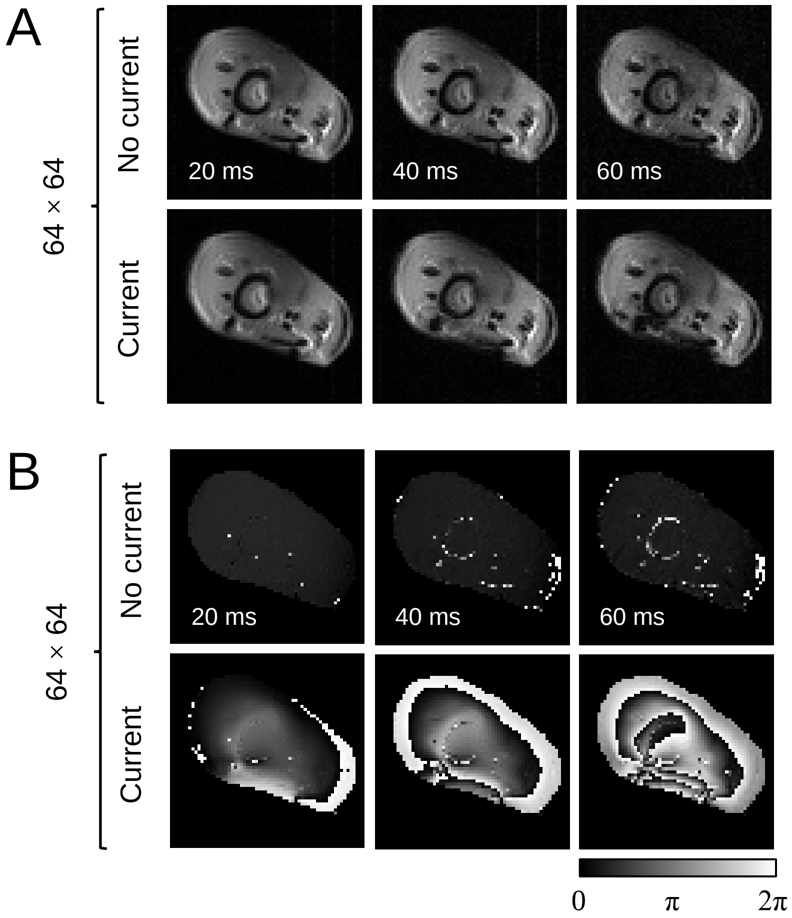

FIGURE S4. Signal magnitude A) and the corresponding signal phase B) images of the 4 mm thick central slice in the transversal orientation across the lower chicken thigh. Slice orientation was also perpendicular to the static magnetic field and the electrodes. The images were acquired with the spin-echo imaging sequence using synchronized electric pulses (Figure 1E) at current injection times t = 20, 40, 60 ms (also equal to the spin-echo time TE), imaging matrix 64×64, and in two states of the sample: with current (lower rows) and without it (upper rows). All the images were acquired at the FOV of 30 mm so that the voxel sizes (along *xy*-direction) were equal to L = 469 μm. Images of the sample with current have significantly less signal in regions with a higher injected current (current-time products), i.e., in proximity of the electrodes and the region between them where current density is higher and with longer times (t = 40, 60 ms). In phase images, these regions coincide with the regions of a higher phase gradient.

3 Comparison of CDI and SMD current detection methods

Main difference between current density imaging (CDI) and signal magnitude decay (SMD) current detection method is that CDI is phase-based while SMD is magnitude-based. This difference in nature of the detection principle has another interesting consequence, namely, phase shift is a linear effect as it is linearly proportional to the injected current, while the signal magnitude decay is a quadratic effect. As seen from Equation 9, it is proportional to the injected current (current-time product) squared. Therefore, advantage of CDI over SMD is the highest at lower injected currents, while this advantage decreases with higher injected currents. Important difference between CDI and SMD detection methods is also that CDI enables direct measurement of the magnetic field B_{c_z} (magnetic field created by the currents) so that its gradient can be calculated with all the spatial components in postprocessing, while SMD enables measurement of only magnitude of this gradient and thus prevents obtaining individual spatial components of this gradient. The latter also precludes the use of SMD in all the reconstruction methods where individual spatial derivatives of B_{c_z} must be known.

Both methods, CDI and SMD, enable the detection of the magnetic field gradient magnitude $G_c = \left|\nabla B_{c_z}\right|$ so that both of them can be used for the reconstruction of current density using the simplification $j_\perp = 2G_c/\mu_0$ introduced in Equation 4. In this case, it is expected that both of them should produce the same results (neglecting different sensitivities, susceptibility to artefacts …). However, this is no longer true if two different reconstruction methods are used, for example approximate $j_\perp = 2G_c/\mu_0$ by SMD or CDI vs. exact $\vec{j} = \nabla \times \vec{B}_c/\mu_0$ by CDI. In this case, the approximation produces good results in longer cylindrical regions with homogeneous current distribution and only approximate results in the regions of other shapes or where the current distribution is inhomogeneous. Difference between these two reconstruction methods still remains to be investigated in more detail numerically and experimentally by samples of other geometries and current distributions, which is a plan for further studies.

Furthermore, another interesting study would also be a comparison of CDI and SMD, which have different principles of current detection, in reconstruction using the simplified model for current density $j_\perp = 2G_c/\mu_0$. A hint of what might be the result of this comparison is provided by a comparison of magnitude with phase images of the test (Figure S2 vs. Figure S3) and biological sample (Figure S4A vs. Figure S4B). It can be seen that the regions with a higher signal loss in signal magnitude images coincide with the regions of higher phase gradient in signal phase images and there is a positive correlation between these two effects which are the highest in the regions with the highest current injections.

A simpler current reconstruction by $j_\perp = 2G_c/\mu_0$ (Equation 4, inner cylinder) might also be proved useful in conductivity reconstruction as it enables simpler performance of the current density imaging with more different current injections and thus enable compensating the reconstruction error inherent for conductivity imaging to some extent.
